# Supplementary material for: Reversal of Tau-Dependent Cognitive Decay by Blocking Adenosine A1 Receptors: Comparison of Transgenic Mouse Models with Different Levels of Tauopathy
Source: Int J Mol Sci. 2023 May 25;24(11):9260. doi: 10.3390/ijms24119260 (PMC10252552; doi:10.3390/ijms24119260)
Supplement: Supplementary file 1 [file ijms-24-09260-s001.zip › ijms-2317678-supplementary.pdf]

SUPPLEMENTARY MATERIALS

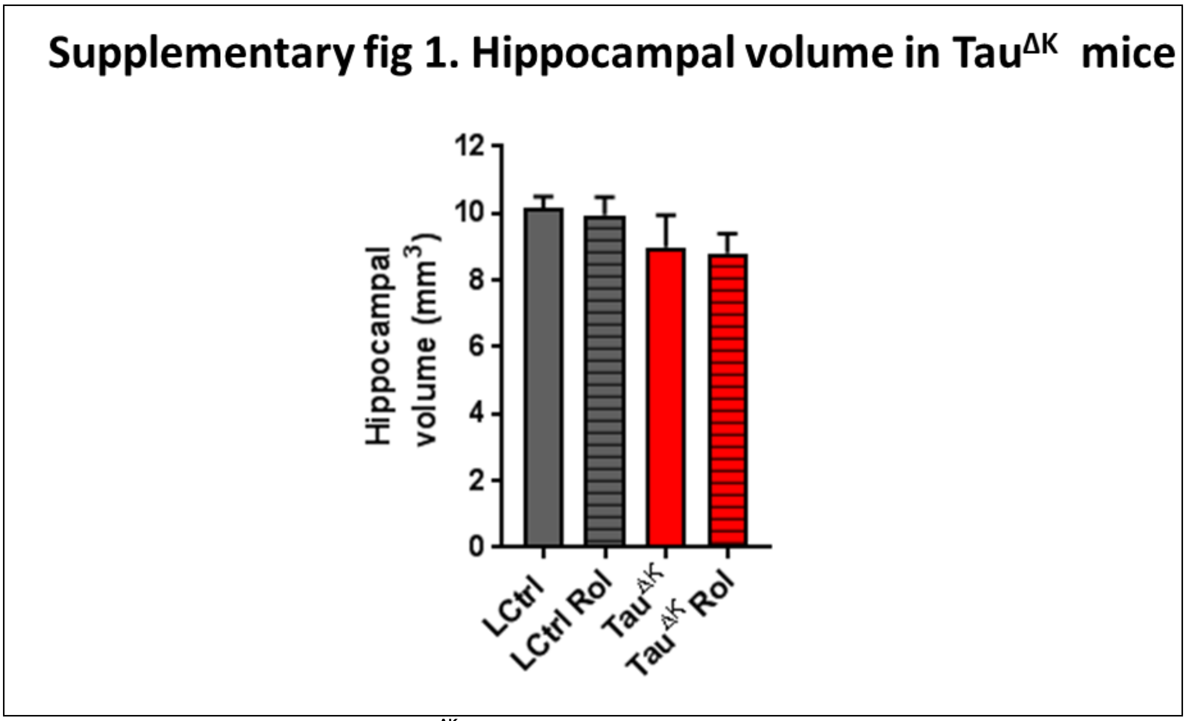

Suppl. Fig. S1: Hippocampal volume in $Tau^{\Delta K}$ mice: No change with rolofylline treatment. Treatment with rolofylline was started at ~15 months. At age ~17.5 months (after 2.5 months of treatment) hippocampal volume of coronal sections was stereologically measured in WT and transgenic mice treated with vehicle or rolofylline. No differences in brain volume were observed. Results are shown a mean ± SEM (n = 5-6/group) and analyzed by one-way ANOVA followed by uncorrected Fisher's LSD test as *post-hoc* test [$F(3,18) = 0.9483$; p=0.4382].

Supplementary figure 2. Training session Fear Conditioning, MWM and Open Field test in tau transgenic animals after rolofylline treatment .

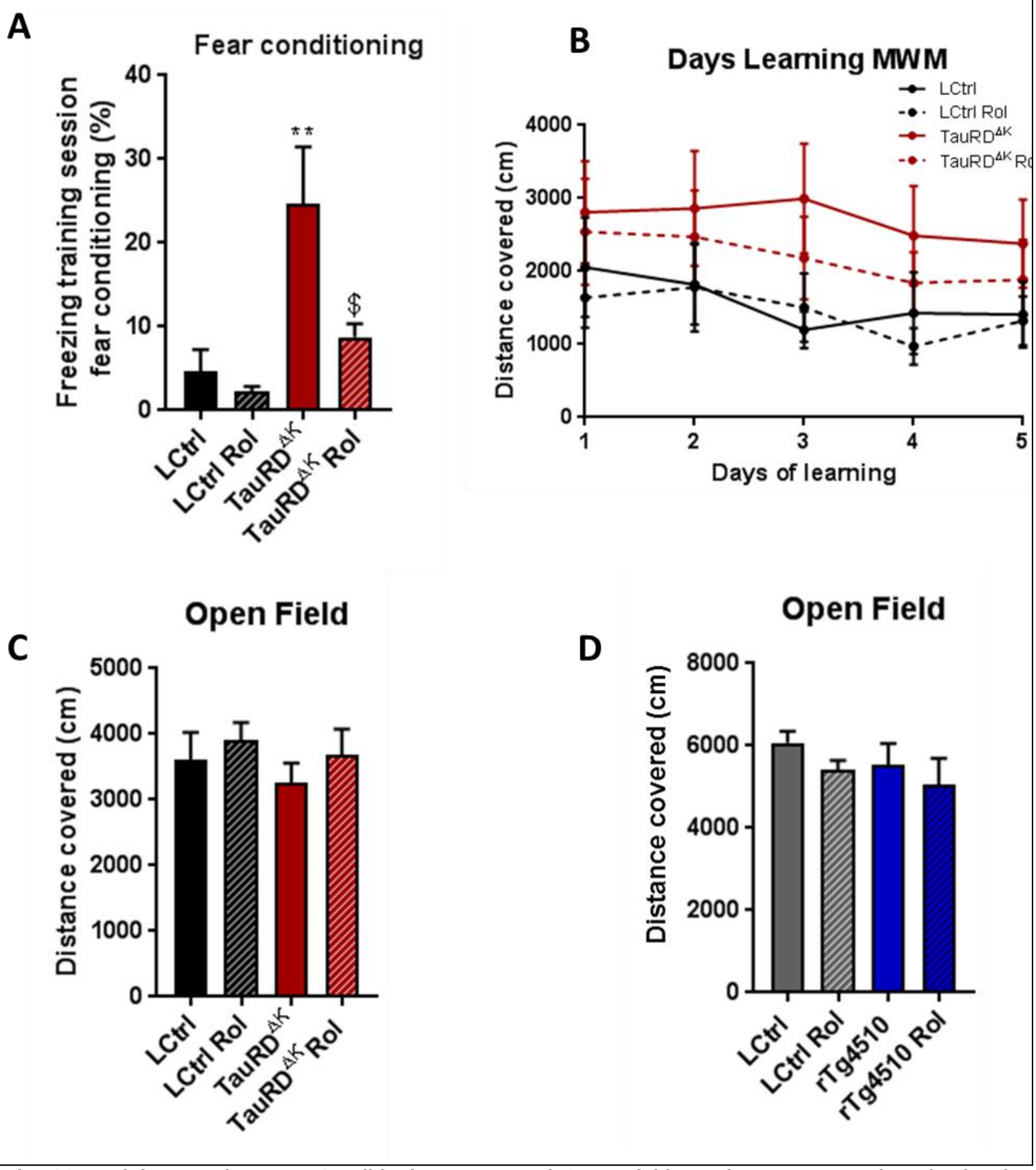

Suppl. Fig. S2: Training session Fear Conditioning, MWM and Open Field test in tau transgenic animals after rolofylline treatment. (A) Graph showing the % of freezing in the training session of fear conditioning in 15 months old animals expressing TauRD$^{\Delta K}$ and their littermate controls treated with rolofylline for 1.5 months. TauRD$^{\Delta K}$ mice showed increased freezing in the training session when compared to LCtrl and TauRD$^{\Delta K}$ Rol treated mice [F(3,35)=7.189, p=0.0007, Tukey´s post-hoc p=0.0082 for TauRD$^{\Delta K}$ vs. LCtrl and p=0.0198 for TauRD$^{\Delta K}$ vs. TauRD$^{\Delta K}$ Rol].

(B) Rolofylline treatment improved learning shown by reduced distance covered of TauRD$^{\Delta K}$ mice in the Morris water maze test (two-way ANOVA F(3,195)=4.871, p=0.0027 for factor "treatment", n=9–13 per group). TauRD$^{\Delta K}$ mice covered more global distance to escape when compared to LCtrl (Tukey´s post-hoc LCtrl vs. TauRD$^{\Delta K}$ mice p=0.0214, *). In contrast, there was no significant difference between LCtrl and TauRD$^{\Delta K}$ Rol (p=0.3549 analyzing mean of all time points).

Graph showing the mean covered distance (in cm) in the Open Field arena during 10 min in **(C)** Fifteen months-old animals expressing in TauRD$^{\Delta K}$ and their littermate controls treated with rolofylline for 1.5 months [F(3,40) = 0.534; *p*=0.6616] and **(D)** rTg4510 and control animals treated with food containing 5 mg/kg of rolofylline at 2.5-3 months of age. No differences are observed between groups. Results are shown a mean ± SEM (n = 9-13/group) and analyzed by one-way ANOVA [F(3,47) = 0.9219; p=0.4376].

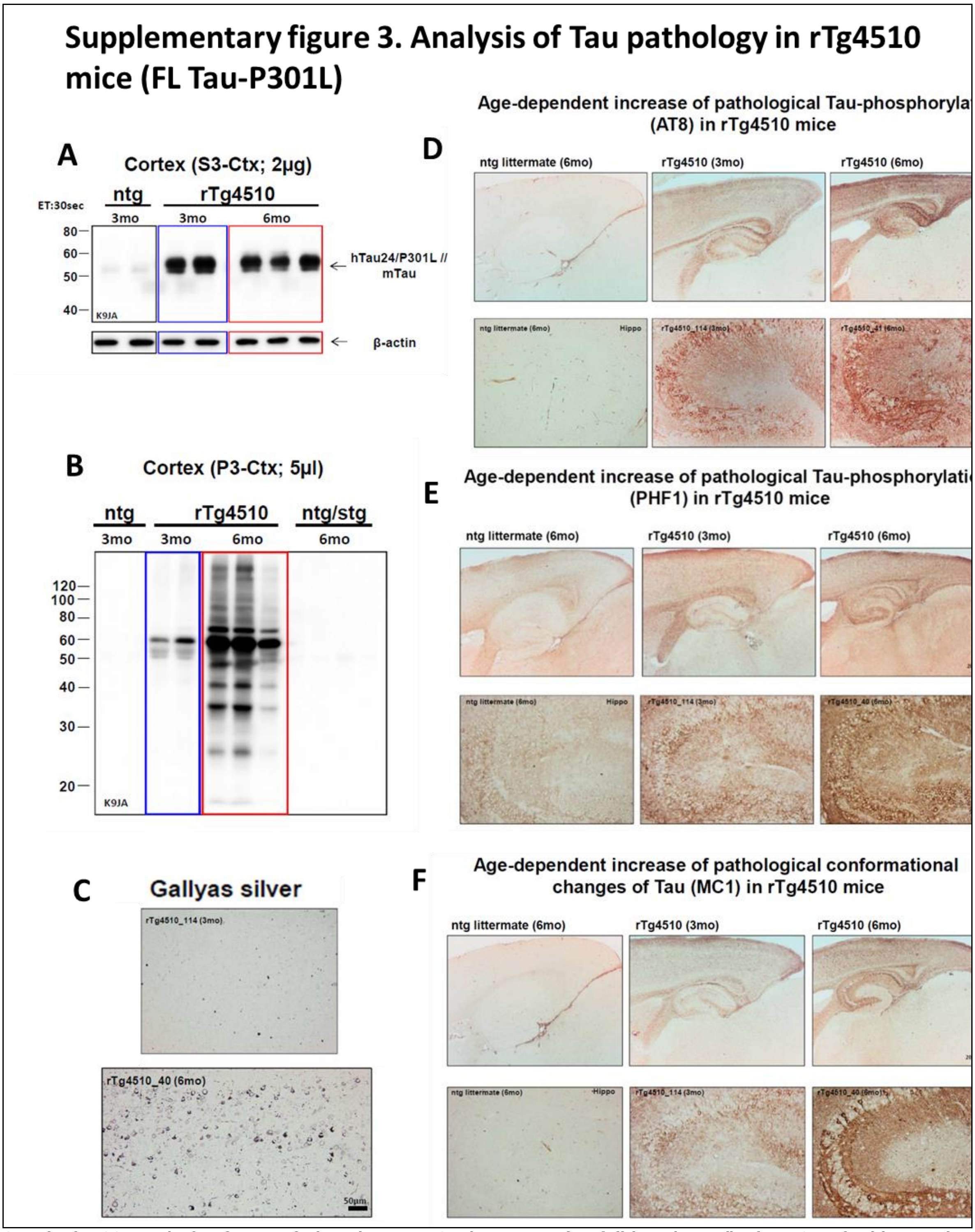

Suppl. Fig. S3: Analysis of tau pathology in rTg4510 mice expressing full length tau (isoform 0N4R) with mutation P301L. Brain tissue was obtained from rTg4510 mice and non-transgenic (ntg) mice at 3 and 6 months of age

(A) Cortical protein levels of human and mouse tau were analyzed by western blot detected by the pan-Tau antibody K9JA. The expression of mutant Tau^{P301L} is high (~13 times endogenous Tau.

(B) Protein levels of insoluble tau species in control and rTg4510 mice 6 and 3 months of age detected by the pan-Tau antibody K9JA. Detergent-insoluble tau appears as early as 3 months of age and increases strongly by 6 months of age. No detergent-insoluble tau was present in the non-transgenic samples.

(C) Gallyas silver staining of tau aggregates become pronounced at ~6 months in the cortex of rTg4510 mice. Scale bar: 50 µm.

(D-F) Histological analysis of conformational changed and phosphorylated tau in rTg4510 mice at 3 and 6 months of age staining with (D) antibody AT8 against phosphorylated tau (dual phosphorylation epitope Ser202+Thr205), **(E)** antibody PHF1 (dual phosphorylation epitope Ser396+Ser404) and **(F)** antibody MC1 against a pathological tau conformation (epitope 5-15 + 312-322, (Jicha et al. 1997). Scale bars: 200 µm.

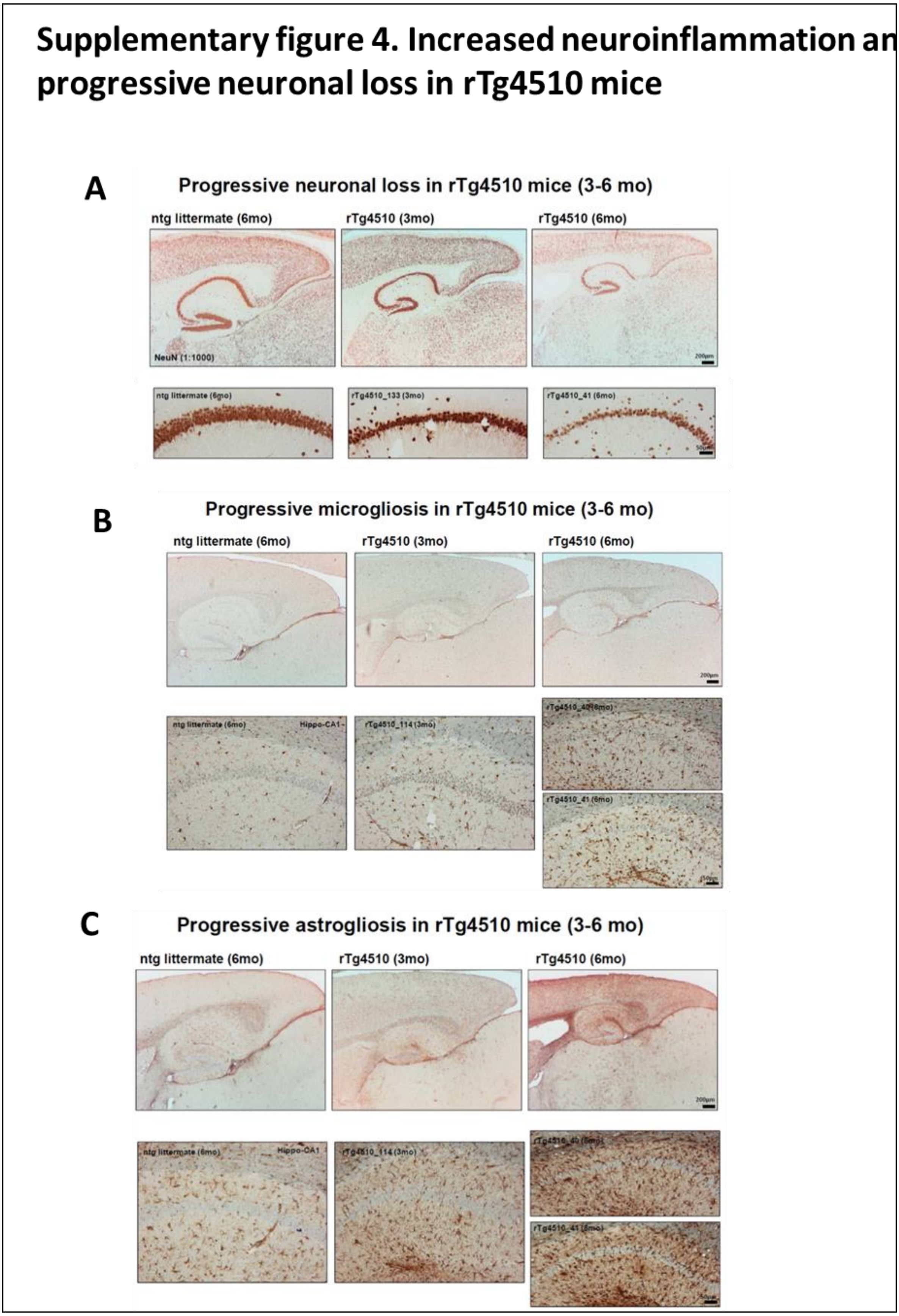

Suppl. Fig. S4: Increased neuroinflammation and progressive neuronal loss in rTg4510 mice. Brain tissue was obtained from rTg4510 and non-transgenic (ntg) mice at 3 and 6 months of age and analyzed by histology staining.

(A) NeuN, a specific neuronal marker, shows an age-dependent increase of neuronal death in the CA1 layer of the hippocampus.

(B) microgliosis and **(C)** astrogliosis were increased as early as 3 months of age (using antibodies Iba1 and GFAP, resp.). Scale bars: 200 µm for overviews in the upper panels and 50 µm for lower panels.

Supplementary figure 5. Comparison of Tau and TSPO imaging across mouse lines.

A

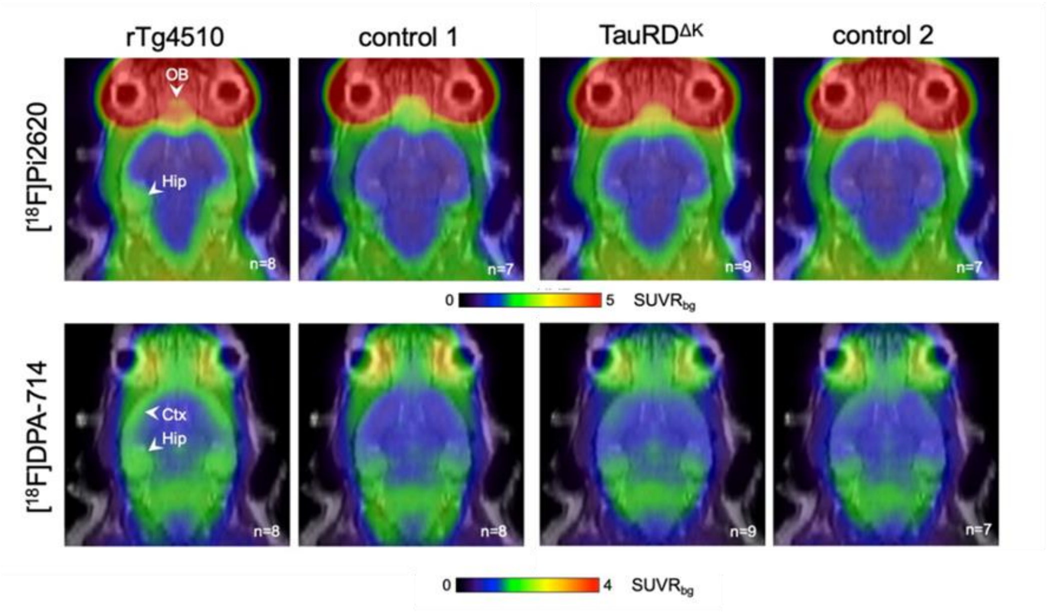

B

Brain area	rTg4510 (n=8)	control 1 (n=7)	TauRD$^{\Delta K}$ (n=9)	control 2 (n=7)	rTg4510 vs ctrl 1	TauRD$^{\Delta K}$ vs ctrl 2	rTg4510 vs TauRD$^{\Delta K}$
Whole brain	1.79 ± 0.15	1.34 ± 0.18	1.57 ± 0.10	1.47 ± 0.21	p=0.9887	p=0.9999	p=0.9999
Olfactory bulb	**4.23 ± 1.50**	2.17 ± 1.21	2.35 ± 0.36	2.18 ± 0.40	**p<0.0001**	p=0.9999	**p<0.0001**
Frontal cortex	**2.55 ± 0.73**	1.33 ± 0.73	1.62 ± 0.50	1.51 ± 0.62	**p=0.0015**	p=0.9999	**p=0.0390**
Anterior cingulate cortex	2.02 ± 0.30	1.17 ± 0.36	1.26 ± 0.17	1.17 ± 0.31	p=0.1751	p=0.9999	p=0.2423
Thalamus	0.83 ± 0.08	0.93 ± 0.14	0.91 ± 0.08	0.81 ± 0.07	p=0.9999	p=0.9999	p=0.9999
Hippocampus	1.09 ± 0.13	0.84 ± 0.09	0.83 ± 0.07	0.73 ± 0.12	p=0.9999	p=0.9999	p=0.9999

C

Brain area	rTg4510 (n=8)	control 1 (n=8)	TauRD$^{\Delta K}$ (n=9)	control 2 (n=7)	rTg4510 vs control 1	TauRD$^{\Delta K}$ vs control 2	rTg4510 vs TauRD$^{\Delta K}$
Whole brain	**1.51 ± 0.13**	1.35 ± 0.07	1.27 ± 0.09	1.25 ± 0.10	**p=0.0334**	p=0.9728	**p=0.0004**
Olfactory bulb	**1.69 ± 0.25**	1.45 ± 0.17	1.44 ± 0.14	1.42 ± 0.11	**p=0.0004**	p=0.9638	**p=0.0001**
Frontal cortex	**1.41 ± 0.23**	1.12 ± 0.09	1.22 ± 0.08	1.16 ± 0.09	**p<0.0001**	p=0.6845	**p=0.0071**
Anterior cingulate cortex	**1.60 ± 0.15**	1.24 ± 0.11	1.19 ± 0.09	1.22 ± 0.11	**p<0.0001**	p=0.9570	**p=0.0004**
Thalamus	1.13 ± 0.08	1.11 ± 0.06	1.20 ± 0.10	1.14 ± 0.07	p=0.9783	p=0.7764	p=0.6407
Hippocampus	**1.37 ± 0.12**	1.19 ± 0.06	1.23 ± 0.08	1.17 ± 0.06	**p=0.0147**	p=0.7887	p=0.0585

Suppl. Fig. S5: Comparison of tau and TSPO imaging across mouse lines. **(A)** Upper row: PET imaging of tau NFTs with the tracer [^{18}F]PI-2620, injected dose ~10 MBq, emission scan 30-60 min p.i. Lower row: PET imaging of TSPO expression in activated microglia (neuroinflammation) using the tracer [^{18}F]DPA-714, injected dose ~10 MBq, emission scan 0-30 min p.i. Displayed are average images, the number of contributing animals is indicated. Image intensity is normalized to background, i.e. tracer-negative areas of the midbrain. Note that rTg4510 mice show NFTs in frontal cortex and olfactory bulb, and pronounced inflammation in cortex and hippocampus. No NFTs are observed in TauRD$^{\Delta K}$ mice with this tracer. **(B)** Quantification of PET imaging of tau NFTs with the tracer [^{18}F]PI-2620 (Upper panels). Two-way ANOVA of [^{18}F]PI-2620 uptake (tau accumulation), followed by Tukey's multiple comparison test. Shown are mean values ± SD of SUVRbg and results of post-hoc testing. Main effects: Factor "brain area": $F(5,135)=71.2$; $p<0.0001$. Factor "group": $F(3,27)=13.1$; $p<0.0001$. **(C)** Quantification of PET imaging of tau NFTs with the tracer [^{18}F]PI-2620 (Lower panels). Two-way ANOVA of [^{18}F]DPA-714 uptake (TSPO expression; inflammation), followed by Tukey's multiple comparison test. Shown are mean values ± SD of SUVRbg and results of post-hoc testing. Main effects: Factor "brain area": $F(5,140)=79.4$; $p<0.0001$. Factor "group": $F(3,28)=9.4$; $p=0.0002$.Control 1: non-transgenic littermates of rTg4510 mice. Control 2: non-transgenic littermates of TauRD$^{\Delta K}$ mice. Abbreviations: Ctx: cortex; Hip: hippocampus; OB: olfactory bulb.
